# Preserved Adrenal Function After Lumbar Spinal Cord Transection Augments Low Pressure Bladder Activity in the Rat

**DOI:** 10.3389/fphys.2018.01239

**Published:** 2018-09-03

**Authors:** Diana V. Hunter, Seth D. Holland, Matt S. Ramer

**Affiliations:** ^1^International Collaboration on Repair Discoveries, Faculty of Medicine, The University of British Columbia, Vancouver, BC, Canada; ^2^International Collaboration on Repair Discoveries, Department of Zoology, Faculty of Science, The University of British Columbia, Vancouver, BC, Canada

**Keywords:** spinal cord injury, bladder, adrenal gland, catecholamines, non-voiding contractions

## Abstract

Spinal cord injury (SCI) disconnects supraspinal micturition centers from the lower urinary tract resulting in immediate and long-term changes in bladder structure and function. While cervical and high thoracic SCI have a greater range of systemic effects, clinical data suggest that those with lower (suprasacral) injuries develop poorer bladder outcomes. Here we assess the impact of SCI level on acute changes in bladder activity. We used two SCI models, T3 and L2 complete transections in male Wistar rats, and compared bladder pressure fluctuations to those of naïve and bladder-denervated animals. By 2 days after L2 transection, but not T3 transection or bladder denervation, small amplitude rhythmic contractions (1 mmHg, 0.06 Hz) were present at low intravesical pressures (<6 mmHg); these were still present 1 month following injury, and at 3 months, bladders from L2 SCI animals were significantly larger than those from T3 SCI or naïve animals. Low-pressure contractions were unaffected by blocking ganglionic signaling or bladder denervation at the time of measurements. L2 (and sham surgery) but not T3 transection preserves supraspinal adrenal control, and by ELISA we show lower plasma adrenal catecholamine concentration in the latter. When an adrenalectomy preceded the L2 transection, the aberrant low-pressure contractions more closely resembled those after T3 transection, indicating that the increased bladder activity after lumbar SCI is mediated by preserved adrenal function. Since ongoing low-pressure contractions may condition the detrusor and exacerbate detrusor-sphincter dyssynergia, moderating bladder catecholamine signaling may be a clinically viable intervention strategy.

## Introduction

Voluntary control of the urinary bladder relies on the coordination of autonomic, sensory and somatic nervous systems (Yoshimura and de Groat, 1997). When supraspinal input to these systems is interrupted by spinal cord injury (SCI) there are immediate and long-term consequences to bladder function. The loss of coordination between the cord-mediated reflex centers that control contraction of the detrusor and the external urethral sphincter often leads to the development of detrusor-sphincter-dyssynergia (DSD), where the bladder and sphincter contract together, resulting in high intravesical pressures and inefficient voiding (de Groat and Yoshimura, 2006). Without intervention, these complications can lead to vesicoureteral reflux, urinary tract infections, and upper urinary tract damage, all of which impact health care costs and quality of life for individuals with SCI (Al Taweel and Seyam, 2015). It is no surprise, then, that advances in bladder management and care are top priorities of both researchers and individuals with SCI (Anderson, 2004).

During normal urinary bladder filling, low-amplitude rhythmic increases of intravesical pressure occur without inducing micturition. These non-voiding contractions (NVCs) have been documented in many animal species, dating back to the time of Sherrington’s experiments in cats, where during bladder filling NVCs preceded higher amplitude voiding contractions (Sherrington, 1892; Streng et al., 2006; Vahabi and Drake, 2015). Recently, the presence of NVCs during normal bladder filling has been linked to activation of primary afferents and may be important for signaling bladder fullness (Iijima et al., 2009; Heppner et al., 2016). However, both denervated and *ex vivo* bladders also exhibit a pattern of increased contractions with filling, indicating that NVCs are likely an intrinsic property of the bladder muscle (Drake et al., 2003; Lagou et al., 2004; Heppner et al., 2016). Importantly, aberrant NVC patterns after SCI are linked to overactivity in the detrusor muscle and changes to primary afferent signaling, which contribute to bladder dysfunction (Kruse et al., 1993; Cheng et al., 1995).

After SCI in both humans and rats, there is an initial loss of voiding reflex that is followed by variable recovery dependent on the level and severity of injury (Pikov et al., 1998; David and Steward, 2010; Ozsoy et al., 2012). Loss of coordination afforded by descending control combined with reorganization of micturition circuits after SCI results in the development of what is termed the neurogenic bladder. DSD and bladder overactivity tend to emerge over time and are widely considered to underlie the hypertrophy of the detrusor muscle. However, during the earliest time points post-injury, while the bladder is still considered areflexic, significant changes occur to the bladder wall (Mimata et al., 1993; Apodaca et al., 2003). In rat spinal cord transection models, the urothelium of the bladder is disrupted within hours of the injury and wet bladder weights and intravesical volumes increase significantly during the first week (Mimata et al., 1993; Apodaca et al., 2003). These findings suggest that dyssynergias, which are prevalent at higher intravesical pressures, may not be the sole cause of structural and functional changes within the bladder wall.

Clinically, bladder outcomes vary with the level of SCI; one study identified patients with acute low-thoracic or high-lumbar SCI as having a greater incidence of vesicoureteral reflux, indicating that lower injuries may result in more significant dysfunction (Kaplan et al., 1991; Suzuki and Ushiyama, 2001). In rats however, most studies investigating bladder activity are performed using a mid to low thoracic injury, and after reflex voiding reemerges (Yoshiyama and de Groat, 2002; Ikeda and Kanai, 2008; Iijima et al., 2009; Hou et al., 2016). This leaves questions with regards to bladder activity in the acute time periods after SCI and the role that the level of SCI plays in their manifestation.

Here we assess bladder activity acutely after SCI by measuring changes in the intravesical pressure of rats with either high thoracic (T3x) or lumbar (L2x) spinal cord transection. We find that within 2 days of L2x there is increased bladder activity at low pressures that is absent 2 days after T3x, and by 3 months post-SCI, bladders from L2x animals are substantially larger than those of T3x rats. We also show that this differential bladder activity is not due to aberrant neural activity, but is dependent on preserved adrenal function after L2x. We suggest that increased adrenal-endocrine mediated activity in the bladder at low-pressures contributes to poorer bladder outcomes when descending control of adrenal function is preserved.

## Materials and Methods

### Animals

The experiment was conducted on 89 adult male Wistar rats (300–400 g, Envigo, Mississauga, ON, Canada). Animals were acclimatized for at least 1 week before surgical procedures and testing in temperature and light-controlled (12 h light–dark reversed cycle) rooms. All surgical and euthanasia procedures were performed in accordance with ethical guidelines of the Canadian Council for Animal Care, with ethical approval obtained from the University of British Columbia. From an additional 28 rats we harvested and weighed bladders 3 months following complete T3 SCI (*n* = 8), L2 SCI (*n* = 9) and naïve rats (*n* = 11), which were surplus to another experiment in our center.

### Intravesical Recordings

Initial recordings at low intravesical pressures were recorded in naïve rats (*n* = 4) and rats 2 days post-transection (T3x *n* = 5; L2x *n* = 5) and 1 month post-sham or transection (L2 sham *n* = 6; L2x *n* = 6). Bladder pressure recordings in awake animals were performed 2 days post-L2x (*n* = 3) and in age matched naïve (*n* = 4) animals. For animals that received a treatment or injury at the time of recordings four animals were used in each group (L2x at time *n* = 4; Hexamethonium bromide, HexBr *n* = 4; pelvic ganglionectomy, PGx *n* = 4). Plasma epinephrine and norepinephrine measurements were performed in animals 2 days post-injury that did not have any other experimental manipulations (T3x *n* = 5; L2x *n* = 5). To assess the response of the bladder to filling, infusions were performed in naïve animals and 2 days after each injury type (naïve *n* = 8; PGx *n* = 5; T3x *n* = 7; L2x *n* = 7; adrenalectomy, AdxL2x *n* = 7).

### Spinal Cord Injury Surgeries

Complete transection of the spinal cord was performed at either the third thoracic (T3) or second lumbar (L2) spinal cord segment in adult male Wistar rats. Sham surgeries were performed concurrently, and in the same manner as the transection surgeries, up to and including the durotomy. Rats were housed in groups of 2–4 at a secure conventional facility with a standard reversed 12 h light-dark cycle. Though details of the surgical procedure and post-operative care have been previously detailed (Ramsey et al., 2010), major procedures will be detailed here.

Prophylactic enrofloxacin (Baytril; 10 mg/kg, s.c., AVP, Langley, BC, Canada) was administered to all surgical animals for 3 days prior to as well as on the day of SCI surgery. The surgeries were performed under 2–2.5% isoflurane (Fresenius Kabi Canada Ltd., Richmond Hill, ON, Canada) in oxygen, maintaining surgical plane of anesthesia throughout the operation. Buprenorphine (Temgesic^®^; 0.02 mg/kg, s.c., University of McGill Animal Resources Centre, Montreal, QC, Canada) and ketoprofen (Anafen^®^, 5 mg/kg, s.c., AVP, Langley, BC, Canada) were administered just prior to surgery for analgesic purposes. The skin at the surgical site was shaved, cleaned three times with alternating hibitane and 70% ethanol, and treated with iodine. The anesthetized animal was placed prone on a water-circulating heating pad for the duration of the procedure.

For T3 transections (T3x) and shams a 5 ml syringe was placed behind the elbows and under the rib cage to reduce curvature of the spine. The spinal column was exposed *via* a midline incision in the skin, approximately 3 cm long above C8-T3 vertebrae. The T2-T3 intervertebral space was then exposed by blunt dissection of the overlying muscle and connective tissue. L2 shams and transections (L2x) received a midline incision above the T11-L1 vertebrae and muscle was removed surrounding the T13 vertebra. A laminectomy at T13 was performed to expose the underlying L2 cord. The sham operations were completed with the opening of the dura and wound repair. For the complete transections, the cord was cut completely with surgical scissors. Completeness was verified under the surgical microscope by visualization of clear separation and retraction of the two cut stumps of the cord. Upon hemostasis, the muscle and skin were closed with continuous, 4-0 Vicryl (Ethicon, Somerville, NJ, United States) sutures, and interrupted, 4-0 Prolene (Ethicon, Somerville, NJ, United States) sutures, respectively.

### Bilateral Pelvic Ganglionectomy (PGx)

A 2cm midline abdominal incision through the skin and muscle layer was made just rostral to the pubis. The major pelvic ganglia were exposed on the ventolateral surface of the prostate by blunt dissection using sterile cotton swabs. Using microscissors and forceps the body of the pelvic ganglion was detached from the connective tissue on the surface of the prostate and carefully cut from the connecting nerves, avoiding large blood vessels. Any small bleeds were treated with pressure applied by a cotton swab. When both pelvic ganglia were removed, the muscle was sutured with continuous 4-0 Vicryl suture and the skin was closed using 5-0 Vicryl subcuticular suture.

### Bilateral Adrenalectomies and L2 Transections (AdxL2x)

Bilateral removal of the adrenal glands was performed immediately prior to L2 transections in the AdxL2x animal group. Prior to the surgery 5 ml of hypotonic saline (0.45% sodium chloride solution) was given subcutaneously (s.c.) to help maintain blood volume and pressure. The surgical site was prepared as described above and the same analgesics and antibiotics were given. Upon reaching surgical plane with isoflurane anesthetic, a midline incision was made above the T10-L2 vertebrae and the adrenal glands were accessed through two small incisions in the muscle layer on either side of the spinal column immediately caudal to the rib cage. The adrenal glands were identified in the retroperitoneal fat pad above the kidneys and carefully excised, minimizing blood loss and tissue damage. The muscle wall was closed with 4-0 vicryl suture. Upon completion of the adrenalectomy, the L2 complete transection was performed as described above prior to suturing the single skin incision.

### Post-Operative Animal Care

Upon completion of surgery, fluids were replaced with warmed Lactated Ringer’s solution (5 ml, s.c.). Animals were allowed to regain consciousness in a warm, temperature-controlled environment (Animal Intensive Care Unit, HotSpot for Birds, Los Angeles, CA, United States). Enrofloxacin (10 mg/kg, s.c.), buprenorphine (0.02 mg/kg, s.c.), ketoprofen (5 mg/kg, s.c.), and warmed lactated ringers (5 ml, s.c.) were administered once per day for 3 days following surgery and thereafter only as needed. Rubber matting was placed under the bedding in the cages of rats with SCI, to assist with traction when moving. To aid in recovery and encourage foraging, water bottles were equipped with long spouts and food was scattered around the cage (Ramsey et al., 2010). Animals were supported with an enriched diet, including Rodent Liquid Diet (Bio-Serv, Flemington, NJ, United States), flavored jelly, breakfast cereals and commercially available rat treats. The bladder was manually expressed 3–4 times daily. Monitoring of body weight, activity level, social behavior, bladder function, healing at the surgery site and clinical signs of morbidity was completed daily for the first 2 weeks after surgery and every 2 days thereafter.

### Bladder Pressure Recordings in Anaesthetized Animals

Rats were given 1.2 g/kg urethane (0.5 g/ml in saline, intraperitoneally (i.p.); U2500, Sigma-Aldrich, St. Louis, MO, United States) to anesthetize to surgical plane, upon which they were placed supine on a water circulating heating pad. A midline abdominal incision was made to expose the detrusor. A purse-string suture (6-0 silk) in the dome of the bladder was made to secure both the pressure transducer end of a Millar Rat Pressure Telemeter (model: TRM54P, Millar, Houston, TX, United States) and a 23 gauge winged infusion line attached to a syringe infusion pump 22 (Harvard Apparatus, South Natick, MA, United States). The pressure transducer of the telemetry device was calibrated using a sphygmomanometer prior to each set of measurements. After confirming the patency of the infusion line, the bladder was emptied via urine withdrawal through the infusion line. To measure changes in pressure within the bladder during filling, room temperature saline was infused into the bladder at a physiologically relevant rate of 100 μl/min (Pollock et al., 1986). Intravesical bladder pressure data were transmitted from the telemeter to a SmartPad (model: TR181, Millar, Houston, TX, United States) connected to a PowerLab 16/30 (model: ML880, ADInstruments, Colorado Springs, CO, United States) and recorded in LabChart v.7 (ADInstruments, Colorado Springs, CO, United States) at 1000 Hz.

### Bladder Pressure Recordings in Awake Animals

Animals (*n* = 4 naïve, *n* = 3 L2x) were anesthetized to surgical plane with 2% isoflurane in oxygen. The pressure transducer was inserted into the bladder and secured with a purse string suture as above. The abdominal muscle wall was then sutured and a pocket for the body of the telemetry device was made by separating the skin from the underlying muscle. Once the telemetry device was tucked under the skin of the abdomen, the skin incision was closed (4-0 prolene, discontinuous sutures). The animal was then allowed to awaken and was placed in an observation cage and continuously monitored for 4 h, when they subsequently reached the experimental endpoint. Measurements were recorded using the same pressure transducer system as in the anesthetized rats during natural filing of the bladder. Since behavioral movements result in changes to the abdominal pressures which can mask changes in bladder pressure, only recordings from resting periods were used for analysis (Streng et al., 2006).

### L2x During Bladder Pressure Recordings

Naïve rats were prepared as above for the anesthetized bladder measurements. Upon completing two infusion cycles, the rats were carefully placed on their stomach and an L2 transection was performed as described above. Upon completion of the transection the animals were returned to the supine position and bladder measurements continued for at least 2 h post-L2x.

### HexBr and PGx During Bladder Pressure Recordings

A subset of rats 2 days post-L2x had pressure recordings taken before and after either hexamethonium bromide (HexBr) injection (*n* = 4) at 20 mg/kg via tail vein injection, or bilateral pelvic ganglionectomy (*n* = 4, performed as described above). The NVC amplitudes were recorded before and 1 h after the intervention at comparable low pressures.

### Pressure Recordings Analysis

**FIGURE 1:**
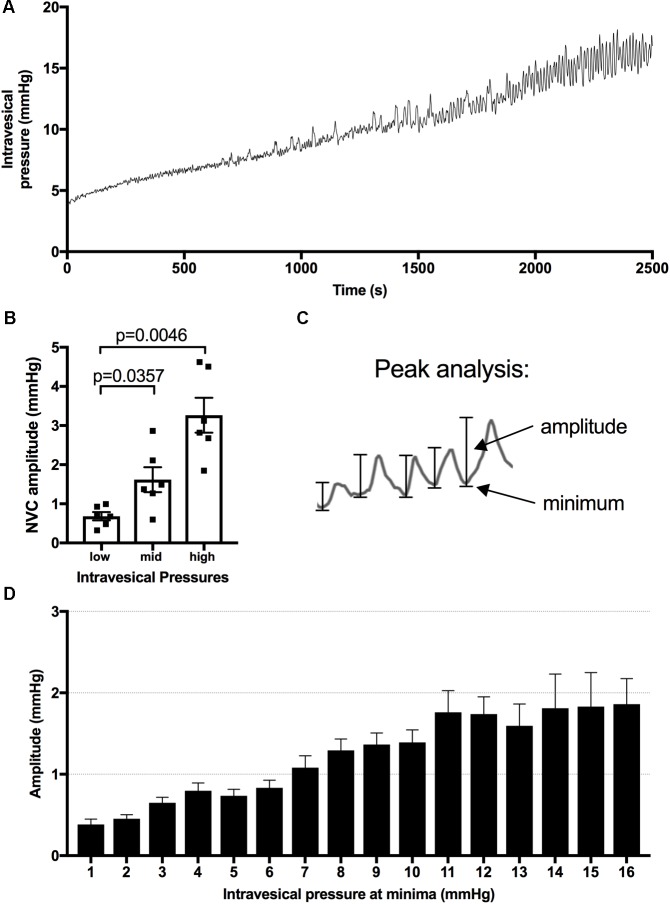
Pressure changes during infusions and analysis of minima and amplitude of bladder contractions. **(A)** Representative pressure recording from a urethane anesthetized rat during bladder filling at a rate of 50 μl/min. **(B)** Amplitude of NVCs are significantly higher at mid (7–10 mmHg) and high (14–18 mmHg) vs. low (1–4 mmHg) intravesical pressure, mean ± SEM, repeated measures one-way ANOVA, *p*-values as shown. **(C)** Peak analysis is used to analyze the relationship between intravesical pressure and NVC amplitude. **(D)** The measured amplitudes are binned based on the intravesical pressure at their minima, the resulting bar graph for naïve animals is presented as an example.

Identification and analysis of NVCs was performed using the Peak Analysis Add-On for Labchart v.7. Collected pressure data was initially smoothed (2 s) and then analyzed under the general unstimulated section in Peak Analysis, with the following parameters: 20 s normalization and minimum two standard deviations between peaks and minimum baseline value between peaks (Heppner et al., 2016). For the comparison of NVC amplitude with increasing intravesical pressure, NVC amplitudes were sorted into 1 mmHg bins based on the minimum starting pressure before each recorded peak (**Figure 1**). A minimum of two infusion cycles were used for each animal in the analysis. Bins with fewer than three NVC amplitudes were not included in the analysis. The intravesical pressure at which the NVC amplitudes reached 1 mmHg were recorded during the bladder infusions and the values were averaged for at least two infusions per animal.

### Epinephrine and Norepinephrine ELISAs

Rats with either an L2 sham, L2x or T3x (*n* = 5 per group) were deeply anesthetized 2 days after injury with 5% inhalant isoflurane and blood was collected via cardiac puncture into K2 EDTA blood collection tubes (BD Vacutainer^®^, Becton, Dickinson and Company, Franklin Lakes, NJ, United States). The animals were immediately euthanized following blood collection. Blood samples were spun at 4°C for 10 min at 1000 ×*g* and plasma was collected and stored at -80°C. ELISAs were performed to measure plasma concentrations of epinephrine (BA E-6100, LDN^®^ immunoassays and services, Nordhorn, Germany) and norepinephrine (CEA907DGe, Cloud-Clone Corp., Katy, TX, United States), following manufacturer’s instructions. For the norepinephrine measurements, the ELISA was performed on only four T3x and L2x animals due to limited plasma volumes. For the final analysis, L2x and L2x sham animals (with preserved descending adrenal control, “spared”) were pooled and compared to those with T3x (without descending control, “lost”). To find the effect on total adrenal catecholamines, we normalized and combined the epinephrine and NE values for each animal. To normalize the measurements, the plasma concentration from each animal was divided by the average “spared” value for the corresponding catecholamine. Normalized epinephrine and norepinephrine values were then averaged.

### Statistical Analyses

All statistical analyses were performed using Graph Pad Prism 7, except in one case (Welch’s ANOVA for unequal variances followed by Games Howell post-test for unequal variances of bladder weights) where we used Real Statistics Using Excel (Excel add-in) ^1^. Statistical tests used and exact *p*-values are given in the results and figure legends. Statistical significance was set at *p* < 0.05. Data are displayed as means ± SEM.

## Results

Patients with acute SCI between T10 and L2 have a greater incidence of vesicoureteral reflux than those with higher-level injuries, suggesting increased bladder hyperactivity after low injury (Suzuki and Ushiyama, 2001). Bladder hyperreactivity and DSD probably both contribute to hypertrophy. The fact that bladder hypertrophy is a gradual process, and that the higher incidence of vesicoureteral reflux in patients with acute low SCI was not correlated with differences in bladder cystometric or biomechanical findings (Suzuki and Ushiyama, 2001), prompted us to ask whether acute changes in bladder activity differed between high and low SCI.

### Short and Long-Term Consequences of High vs. Low SCI on the Rat Bladder

**FIGURE 2:**
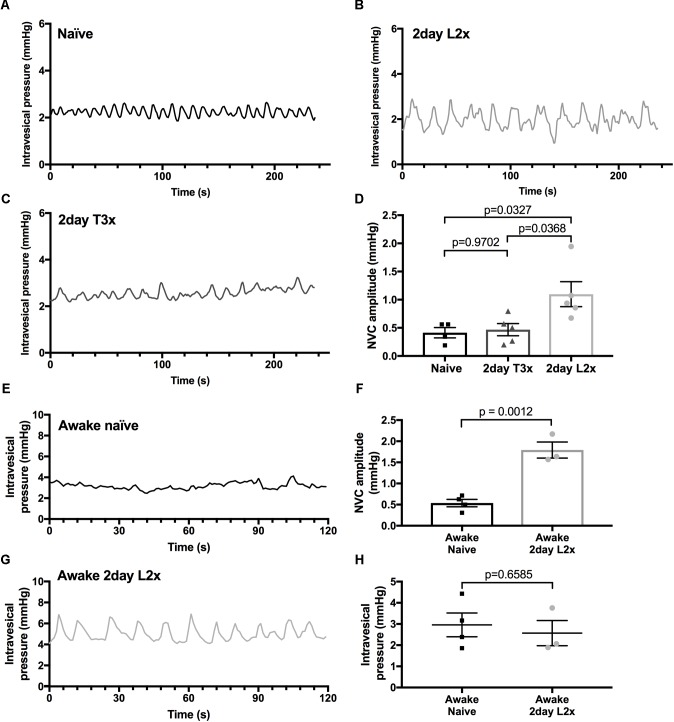
Amplitude of NVCs is increased at low pressures after L2x. **(A–C)** Representative traces of intravesical bladder pressures after different injury levels in urethane anesthetized rats. **(D)** Amplitude of NVCs is significantly higher 2 days post-L2 transection (L2x) compared to 2 days post-T3 transection (T3x) and naïve controls; one-way ANOVA, mean ± SEM. **(E,G)** Representative traces of low intravesical bladder pressures of awake rats that are naïve or 2 days post-L2x. **(F)** The amplitude of NVCs in awake L2x animals is significantly higher than that in naïve animals, **(H)** with no difference in baseline intravesical pressures, *t*-tests, mean ± SEM. Exact *p*-values shown.

Consistent with previous studies, infusion of saline into the naïve bladder results in a steady increase in intravesical pressure and the appearance of increasingly greater NVC amplitudes as the pressure within the bladder increases (**Figures 1A,B**; Streng et al., 2006). When we assessed bladder activity at low pressures (<6 mmHg) in rats 2 days post-L2x we found that the amplitude of NVCs was significantly greater than those in naïve rats or those transected at T3 and occurred at a frequency of 0.061 ± 0.0043 Hz (*n* = 12) (**Figures 2A–D**). Since anesthesia is known to impact the micturition response in both naïve and SCI injured rats we wanted to confirm that the presence of these low-pressure, small amplitude NVCs were not due to urethane (Yoshiyama et al., 1994, 2013; Matsuura and Downie, 2000). The amplitude of NVCs in awake L2x animals (**Figure 2E**) was significantly higher than in naïve animals (**Figures 2F,G**) at the same low intravesical pressures (**Figure 2H**).

**FIGURE 3:**
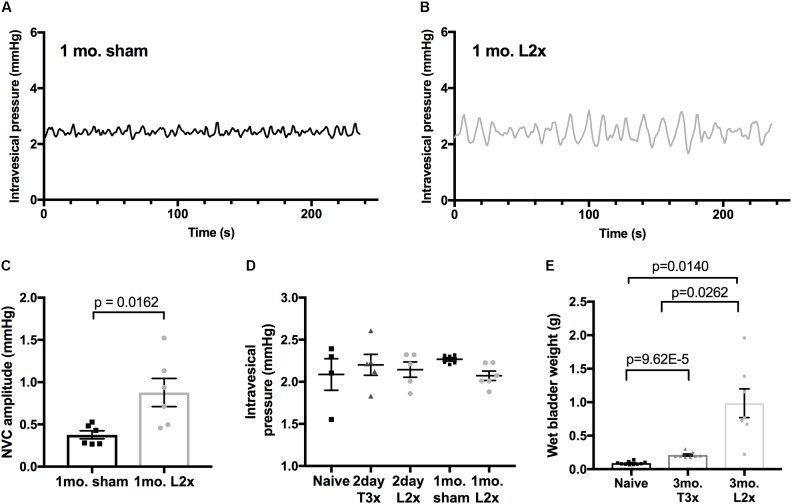
Increased amplitude of low-pressure contractions persist to 1-month post-L2x**. (A–C)** Increased NVC amplitude after L2x is still elevated at least 1 month post-injury compared to sham operated animals; unpaired *t*-test, mean ± SEM. **(D)** The baseline intravesical pressure at which the NVCs were measured at was not different between groups, one-way ANOVA (*p* = 0.5602). **(E)** Wet bladder weights differed significantly by 3 months post SCI (or sham injury) between all groups, one-way Welch’s ANOVA (*p* < 0.0001) followed by Games Howell post-test for unequally distributed data.

To ask whether this phenomenon was restricted to acute time points after L2x, we compared bladder activity in animals 1 month after L2x or sham injury (**Figure 3**). The larger amplitude low-pressure NVCs persisted for at least 1 month following injury (**Figures 3A–C**), and did not depend on differences in baseline intravesical pressures (**Figure 3D**). By 3 months following injury, L2x bladders were significantly heavier than T3x bladders, which were in turn significantly heavier than those from sham-injured rats (**Figure 3E**).

### Increased Low-Pressure NVC Amplitude Emerges in the Acute Period

**FIGURE 4:**
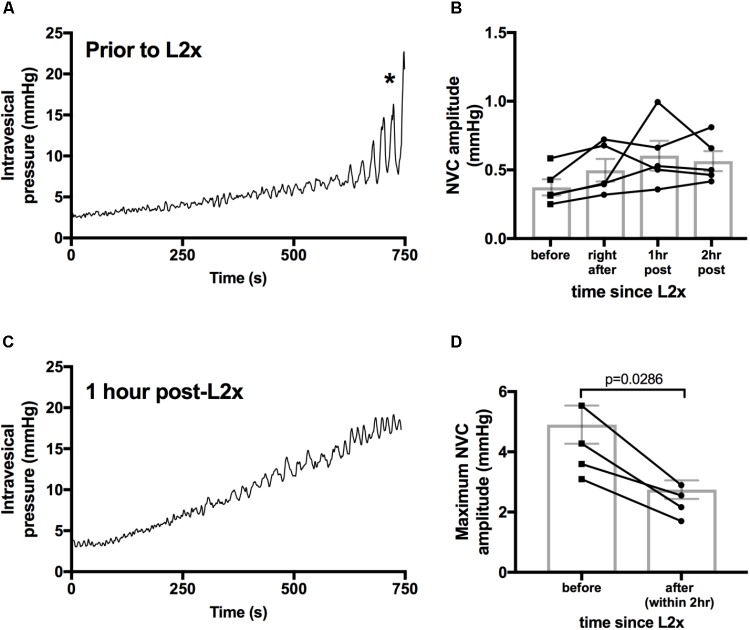
Larger amplitude NVCs at higher pressures are immediately reduced by L2 transection. **(A,C)** Example bladder pressures traces from one animal during 100 μl/min saline infusion either before **(C)** or 1 h after **(D)** L2 transection, ^∗^indicates start of bladder voiding in naïve animal. **(B)** There is no difference in low pressure NVC amplitudes immediately following and up to 2 h after L2x, repeated measures ANOVA, *p* = 0.1939. Lines connect individual animals over the 2 h timeline**. (D)** Maximum amplitude of NVCs during infusions decreased upon transection of the L2 cord, Mann–Whitney two tailed test. Exact *p*-values shown.

Detrusor hyperactivity following SCI has been mainly attributed to loss of descending modulation from supraspinal storage and micturition centers (Samson and Cardenas, 2007). Therefore, we asked whether hyperacute SCI (up to 2 h following transection) led to the emergence of NVCs by interrupting descending control. In these experiments we examined NVC-pressure relationship during bladder filling; amplitudes of NVCs increase and drive afferent activity which is important for sensation of bladder fullness (Heppner et al., 2016). Similar to previous reports, we found that NVC amplitudes increase during physiologically relevant filling of *in vivo* naïve rat bladders under urethane anesthesia (**Figures 1A**, **4A**). Following L2x, NVC amplitude at low pressures remained statistically unchanged for at least 2 h post-injury (**Figure 4B**) indicating that acute loss of descending modulation is not responsible for suppressing ongoing NVC activity. However, immediately following spinal cord transection there is significant decline in the maximum amplitude of NVCs reached at high pressures, which is consistent with these animals’ failure to void (**Figures 4C,D**).

### Increased Low-Pressure NVC Activity Is Not Dependent on Neural Circuitry Below an L2 SCI

**FIGURE 5:**
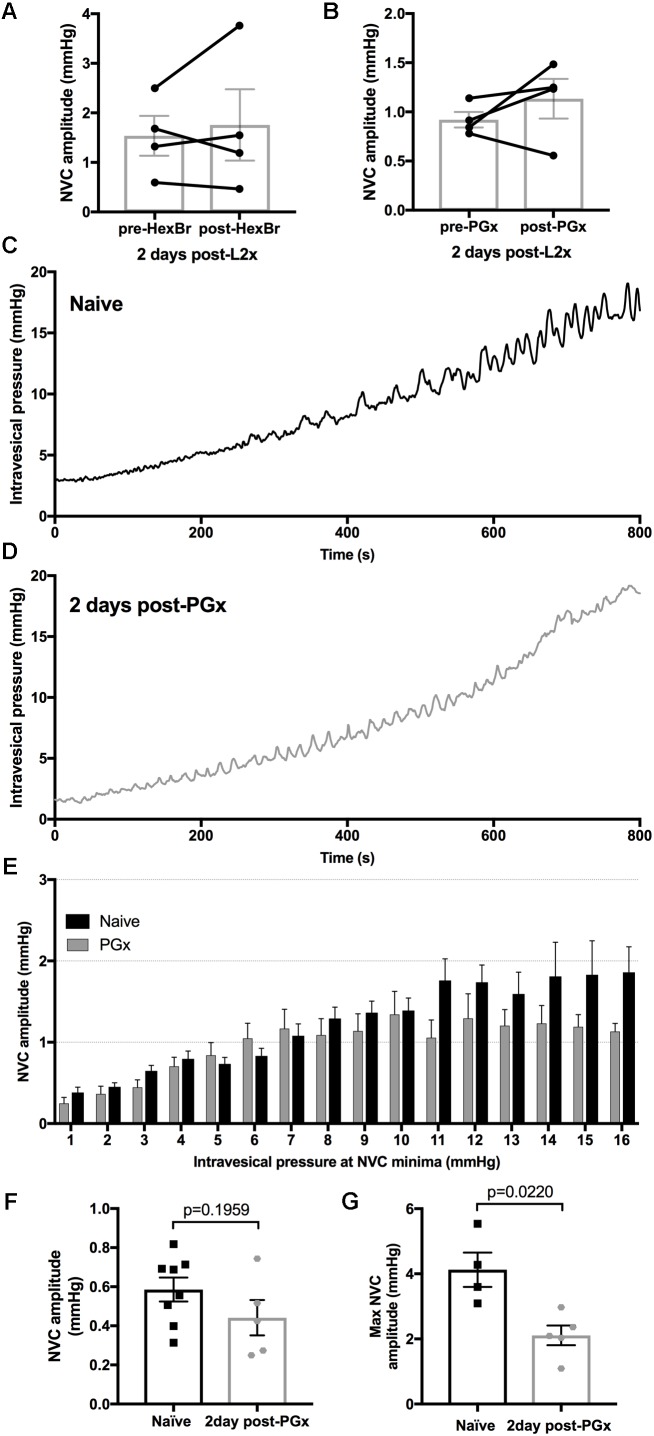
Eliminating bladder innervation results in decreased large amplitude NVCs but does not eliminate the low pressure NVCs 2-days post-L2x. **(A,B)** NVCs from animals 2 days post-L2x were recorded before and after treatment with hexamethonium bromide (HexBr, **A**) or bilateral removal of the pelvic ganglia **(B)**. Neither treatment affected the NVC amplitude at low pressures, paired *t*-tests, **(A)** *p* = 0.6042**; (B)** *p* = 0.3285. **(C,D)** Representative traces of intravesical pressures during bladder filling, 100 μl/min of saline in naïve animals **(C)** and 2 days post-bilateral pelvic ganglionectomy (PGx, **D**). **(E)** NVC amplitudes sorted into 1 mmHg bins based on intravesical pressure at NVC minima (see **Figure 1**). As pressure increases, NVC amplitude increases in naïve animals (**C,E**, *n* = 8) but higher amplitude NVCs are lost when bladder is denervated (**D,E**, *n* = 5). **(F,G)** NVC amplitudes at low pressures are not increased 2 days post-PGx **(F)** but the maximum NVC amplitude reached was significantly lower 2 days post-PGx **(G)**, unpaired *t*-tests, exact *p*-values shown.

Larger NVCs at lower pressures could indicate enhanced sacral parasympathetic output from the cord, possibly driven by amplified bladder-afferent input. To test this hypothesis, we used hexamethonium bromide (HexBr), a ganglionic transmission blocker, to eliminate signals from the cord to the pelvic ganglia in rats 2 days following L2x. HexBr administration at the time of recordings did not reduce low-pressure NVC amplitude (**Figure 5A**), arguing against reflexive neurogenic hyperreflexia either at the level of sacral parasympathetic output, or at the level of thoraco-lumbar sympathetic output (which is partially preserved following L2x).

Primary afferent axons also have efferent functions (such as neurogenic vasodilation), and so we asked whether low-pressure NVCs could be mediated by sensory axons directly (Maggi, 1990). To this end we acutely removed pelvic ganglia (through which bladder afferents travel) bilaterally (PGx) in rats with L2x performed 2 days earlier. Acute PGx did not reduce low-pressure NVC amplitude, arguing against an ongoing efferent role for sensory axons (**Figure 5B**).

We also explored the possibility that the loss of autonomic or efferent-type sensory activity in the detrusor underpinned the emergence of low-pressure NVCs over the first few days following injury. We therefore performed a bilateral PGx 2 days prior to measuring NVC-pressure relationships and compared them to naïve rats (**Figures 5C–G**). By sorting the NVC amplitudes into bins based on the intravesical pressure at the NVC minima (see **Figure 1**) we were able to compare the relationship between increasing intravesical pressure and NVC amplitude (**Figures 5E,F**). The denervation of the bladder resulted in a decrease of maximum NVC amplitude (**Figure 5G**) similar to that of L2x (**Figure 4D**) but did not result in higher NVC amplitudes at low pressures (**Figure 5F**). Therefore, increased NVC amplitude at low pressures following L2x is not due to hypoactivity in de-centralized (parasympathetic), partially preserved (sympathetic), or primary afferent bladder circuitry. NVC frequency did not differ between any of the anesthetized L2x groups (*p* = 0.2916, one-way ANOVA).

### Adrenal Function Underlies Low-Pressure NVCs Following L2 SCI

Previous studies in humans found that individuals with injuries below T5 had significantly higher plasma epinephrine levels compared to individuals with higher injuries (Schmid et al., 1998a,b). A recent study using similar models of high and low SCI in mice identified significant differences in adrenal function between injury groups, which impacted immune function. Mice with high thoracic transections had decreased circulating norepinephrine acutely after injury compared to low injuries and controls (Prüss et al., 2017).

**FIGURE 6:**
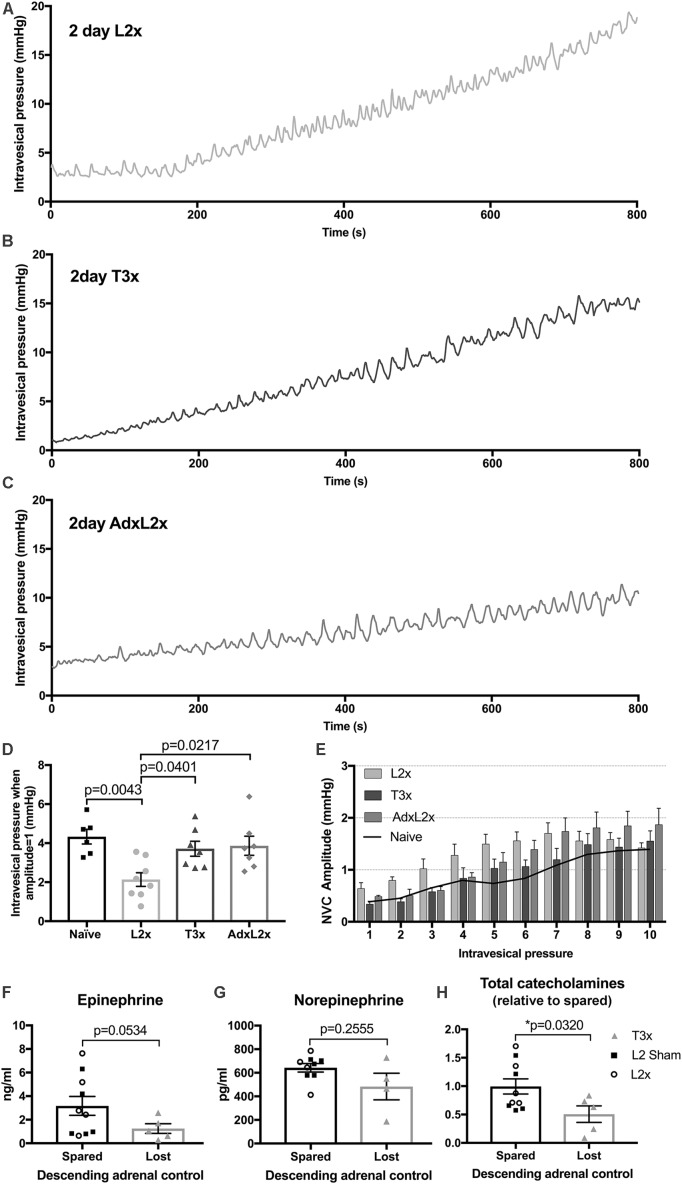
Relationship between amplitude of contractions and intravesical pressure after L2x is governed by adrenal function. **(A–C)** Representative traces of intravesical pressures during 100 μl/min[[Au Query: Please cite “**Figure 6G**” inside the text.]] of saline bladder filling 2-days after **(A)** L2x, **(B)** T3x and **(C)** AdxL2x. **(D)** The intravesical pressures at which the NVC amplitudes reach 1 mmHg for each injury group are plotted, amplitudes reach 1 mmHg at lower pressures 2 days post-L2x compared to naïve, T3x or AdxL2x; one-way ANOVA, Tukey’s multiple comparisons test, mean ± SEM, exact *p*-values shown. **(E)** NVC amplitudes sorted into 1 mmHg bins based on intravesical pressure at NVC minima (see **Figure 1C**) show elevated amplitudes at low pressures after L2x, but not after T3x or when an adrenalectomy is performed immediately prior to the L2x (AdxL2x). Black line in **(E)** shows mean naïve values from **Figure 5E** for reference. **(F,G)** Plasma concentrations of individual adrenal catecholamines were not significantly different between animals with preserved (pooled L2 sham and L2x), or lost (T3x) descending adrenal control 2 days after surgery. **(H)** However, overall relative total adrenal catecholamine concentration was significantly higher in animals with preserved adrenal function than in those with lost descending control (mean ± SEM, unpaired *t*-tests with Welch’s correction, exact *p*-values shown).

To determine whether adrenal function contributed to NVC development 2 days after L2x injury we surgically removed the adrenal glands immediately prior to L2x (AdxL2x). The raw low-pressure bladder traces 2 days post-AdxL2x (**Figure 6C**) more closely resembled those occurring after T3x (**Figure 6B**) than those following L2x alone (**Figure 6A**). NVCs in animals with the L2x alone reached an amplitude of 1 mmHg at lower pressures than they did in naïve, T3x or AdxL2x animals (**Figure 6D**). The distribution of NVC amplitudes at low pressures (0–6 mmHg) in the AdxL2x animals also more closely resembled that of T3x rats than that of L2x animals (**Figure 6E**).

Next we asked whether the contribution of adrenal function to larger NVCs in L2x animals involved increased plasma catecholamine concentration. There was no difference between L2x animals and sham-operated controls for either epinephrine (**Figure 6F**) or norepinephrine (**Figure 6G**), or for total plasma adrenal catecolamine concentration (**Figure 6H**, *t*-tests), indicating preserved adrenal function following L2x. To confirm the previously reported effect of high SCI on circulating catecholamines, we then compared pooled L2x and sham L2x (with preserved adrenal function) with T3x (with lost descending adrenal control). As expected, there was indeed a significant difference between animals with spared descending adrenal function and those without (**Figure 6H**; *p* = 0.0320, Welch’s two-tailed unpaired *t*-test), driven by a drop in concentration of the primary adrenal catecholamine epinephrine (**Figure 6F**; *p* = 0.0534, Welch’s two-tailed unpaired *t*-test).

## Discussion

The development of neurogenic bladder hyperactivity that occurs in the weeks following SCI in the rat is expected and well documented (Kruse et al., 1993; Yoshimura and de Groat, 1997; de Groat and Yoshimura, 2006). However, after L2x we saw an unexpected phenomenon of increased bladder activity, presenting as small amplitude NVCs at low pressures in the acute time period (within 2 days) that was not present after T3x. Examination of both direct and indirect neural pathways led us to link this phenomenon to adrenal gland function, which is better preserved after L2x than T3x.

### Timing of NVC Changes After Transection

Acutely after SCI, during the period of spinal shock when the bladder is areflexic, lower urinary tract activity is present, which is also true of acutely decentralized bladders (Rossier et al., 1979; Heppner et al., 2016). Therefore, even though the spinal cord-mediated micturition reflex is not present, bladder properties can change in a hyperacute timeframe after injury (Apodaca et al., 2003). Changes in the structure of the bladder wall occur within hours after SCI. In rats 2 h after thoracic transection, uroepithelial barrier function is compromised, showing signs of cellular breakdown that lasts up to 2 weeks after injury (Apodaca et al., 2003). Blocking efferent activity at the time of transection was also shown to prevent bladder epithelial disruptions, implicating efferent signaling from the cord in acute bladder dysfunction. However, the delay we see in the development of the increased bladder activity after lumbar SCI is consistent with a slower-acting mechanism, such as might be produced via hormonal changes.

### Interrupting Signaling From Cord to Bladder

Rat pelvic ganglia contain both sympathetic and parasympathetic ganglionic cell bodies as well as primary afferent fibers that innervate the pelvic organs. It has been suggested that the PG could be involved in the integration of pelvic organ signaling, particularly after SCI (Keast, 1999). We initially hypothesized that the differences seen in the L2x group was caused by changes that occur within the PG when the thoraco-lumbar preganglionics and central projections of L1/2 DRG neurons that course through the ganglia are directly damaged. This seemed to be supported by previous studies where bladder epithelial breakdown was prevented by blocking ganglionic transmission (Apodaca et al., 2003). Additionally, a recent study by Persyn et al. found that the at-time removal of the PG resulted in increased baseline bladder pressure and NVC amplitude. This effect did not occur with transection of the hypogastric and pelvic nerves indicating that there may be changes within the PG itself (Persyn et al., 2016). However, in our experiments 2 days after L2x, blocking signals through the PG, either by HexBr treatment or by bilateral pelvic ganglionectomy, did not change the amplitude of the low pressure NVCs. Furthermore, 2 days after the bilateral removal of the PG (in the absence of SCI) there was no increase in NVC amplitude at low pressures. Taken together these results led us to look for an alternative signaling pathway, outside of direct neural connections, that differs between thoracic and lumbar transections.

### Adrenal Medulla Innervation and Function After SCI

Studies that investigate how the level of SCI affects hormone production have shown a difference in adrenal medullary production of epinephrine with high and low SCI: individuals with lower SCIs have higher levels of circulating epinephrine and norepinephrine compared to individuals with higher level injuries (Munro and Robinson, 1960; Schmid et al., 1998a,b, 2000). The adrenal gland is innervated by cholinergic preganglionic sympathetics arising from the intermediolateral column of the T1 to L1 segments in the rat spinal cord, with the majority of the preganglionics residing in T9 and the adjacent segments (Parker et al., 1993). Therefore, T3x eliminates the majority of the circuitry innervating the adrenal preganglionics, whereas L2 transections preserve these connections. This is supported by a recent publication by Prüss et al. that examined the role of adrenal function in sympathetic and immune system function after different levels of spinal cord transection. They found that mice with a high thoracic spinal cord transection had lower norepinephrine levels and poorer immune function compared to those with lumbar transections (Prüss et al., 2017). We also found higher plasma adrenal catecholamine concentrations in animals with low spinal transections, driven primarily by the epinephrine component. Plasma norepinephrine concentration is a function of both sympathetic nerve activity and adrenal function, and so it makes sense that the difference between low and high transection is not as evident. However, when both adrenal catecholamines are considered together, there is a statistically significant decrease in plasma concentration in animals in which descending input to adrenal preganglionic neurons is interrupted.

A potential caveat is that capturing changes to the baseline levels of circulating catecholamines is challenging; for example, epinephrine is produced very rapidly in response to stress (2 s) and the half-life in the blood is very short (less than 70 s); similarly, it is tightly regulated (Goldstein et al., 1983; McCarty, 1983). Therefore, experimental manipulations are likely to cause rapid changes in levels of these catecholamines, increasing the inherent variability; the lower variability in T3x animals supports the idea a loss of descending adrenal control. Notwithstanding the known difficulties in measuring plasma catecholamines, results from the surgical removal of the adrenal gland alongside L2x supports the influence of adrenal signaling on bladder function after SCI.

### Adrenergic Signaling in the Bladder

Two α-adrenergic and three β-adrenergic receptor isoforms are expressed in parts of the lower urinary tract and there is ample evidence that small contractions of the bladder wall are sensitive to adrenergic signaling (Seguchi et al., 1998; Fujimura et al., 1999; Michel and Vrydag, 2006). Indeed, catecholaminergic therapies for overactive bladder generally involve antagonizing alpha receptors (1a/D) or activating beta receptors (beta-3) (Michel, 2015). What effect circulating catecholamines have on the bladder are unknown, but it has previously been speculated that circulating epinephrine may play a role in the activation of adrenergic receptors in the bladder (Michel and Vrydag, 2006). Although still a matter of conjecture, at least two scenarios could account for adrenergically mediated large low-pressure NVCs after SCI. One is that circulating adrenal epinephrine and norepinephrine act on alpha-1 receptors to stimulate contractions, or, that these catecholamines stimulate beta receptors, effecting (over)relaxation and hence stretch-induced contractions via, e.g., the urothelial mechanosensory ion channel Piezo1 (Miyamoto et al., 2014; Michishita et al., 2016). In either case, it can be argued that it is probably not a change in plasma catecholamine concentration which underlies aberrant bladder activity following L2x (there is no evidence that relative concentration differs from sham controls, **Figure 6A**). Rather, it is the effects of preserved adrenal control on the bladder after SCI. That is, reduced plasma catecholamines after high thoracic injuries is protective. The very large differences in bladder weights between T3x and L2x groups, and the smaller differences between sham and T3x groups supports this notion. (**Figure 3E**).

### Adrenal Cortex and the Bladder

Stress related hormones have previously been linked to overactive bladder symptoms. Numerous studies have linked stress, anxiety and depression to lower urinary tract dysfunctions including overactive bladder (Lai et al., 2015). However, it is unclear whether the bladder symptoms are contributing to the stress or the other way around (Lai et al., 2016). Corticotropin-releasing factor (CRF, and ultimately cortisol) has been implicated in social-stress induced bladder dysfunction, including an increase in the number of neurons immunoreactive for CRF in Barrington’s nucleus, an area of the brainstem involved in controlling micturition (Wood et al., 2009). In this study of male rats subjected to daily social stress for 1 week, there were distinct urodynamic functional changes including the presence of NVCs in stressed animals. However, since SCI at any level should preserve the hypothalamic-pituitary-adrenal axis, our results argue in favor of circulating catecholamines from the adrenal medulla as underlying increased low-pressure NVC activity, rather than products of the adrenal cortex.

### Clinical Use of Norepinephrine in the Acute Management of Spinal Cord Injury

Clinical guidelines currently recommend the augmentation mean arterial pressure to 85–90 mm Hg for the first 7 days after an acute SCI (Ryken et al., 2013). This is done to maintain adequate spinal cord perfusion pressure, and is accomplished with the administration of vasopressors (Inoue et al., 2014). No single vasopressor is universally used in the management of SCI but recent evidence suggests that norepinephrine may emerge as the optimal choice (Streijger et al., 2018). The results presented here suggest that this systemic administration of catecholamines to thoracic or cervical spinal cord injuries could adversely impact bladder outcomes. This further highlights the importance of understanding the interdependence of individual physiological systems that are often studied independently after SCI.

In summary, neurogenic bladder is a significant detriment to quality of life after SCI, and those with lower injuries have poorer bladder outcomes due to vesicoureteral reflux and consequent higher chance of kidney dysfunction. In this work, we show that after lumbar transection, even at very low pressures and at acute time points, the bladder undergoes small rhythmic contractions that develop when supraspinal control of the adrenal medulla remains intact. This activity develops early, and is persistent. Such ongoing activity is likely to strengthen the detrusor and thus exacerbate detrusor-sphincter dyssynergia at higher pressures and at more chronic stages. Our data suggest that targeting differences in adrenal medullary function after SCI may impact bladder outcomes. An injury level-dependent influence on the development of dysfunction via peripheral mechanisms is not a new concept: the development of autonomic dysreflexia after high injuries that interrupt neural control of mesenteric arterial bed vascular tone has been linked to a number of changes in the periphery (Ramer et al., 2012). The findings of this research continue to reinforce that the consequences of SCI do not arise merely from direct damage to neural circuits, but also from subsequent and systemic neurogenic and endocrine effects.

## Author Contributions

DH, SH, and MR conceived and designed the study. MR acquired funding and supervised the study. DH and SH performed the surgical procedures and responsible for the animal care. DH acquired and analyzed the data. All authors contributed to the interpretation of the data and to the writing and editing of the manuscript. All authors have approved the final version of the manuscript and agreed to be accountable for all aspects of the work. All persons designated as authors listed are qualified for authorship, and all those who qualify for authorship are listed.

## Conflict of Interest Statement

The authors declare that the research was conducted in the absence of any commercial or financial relationships that could be construed as a potential conflict of interest.

## Footnotes

1 www.real-statistics.com
